# Ginseng (*Panax ginseng* Meyer) Oligopeptides Protect Against Binge Drinking-Induced Liver Damage through Inhibiting Oxidative Stress and Inflammation in Rats

**DOI:** 10.3390/nu10111665

**Published:** 2018-11-04

**Authors:** Rui Liu, Qi-He Chen, Jin-Wei Ren, Bin Sun, Xia-Xia Cai, Di Li, Rui-Xue Mao, Xin Wu, Yong Li

**Affiliations:** 1 Department of Nutrition and Food Hygiene, School of Public Health, Peking University, Beijing 100191, China; lrui_pku@163.com (R.L.); qiheyuntian@163.com (Q.-H.C.); ren_en_jinwei@126.com (J.-W.R.); berseraphim@bjmu.edu.cn(B.S.); caixx1988@ccmu.edu.cn (X.-X.C.); lidiyy@126.com(D.L.); maoruixue@163.com(R.-X.M.); wuxin12@bjmu.edu.cn (X.W.); 2 School of Public Health, Beijing Key Laboratory of Environmental Toxicology, Capital Medical University, Beijing 100069, China

**Keywords:** ginseng oligopeptide, binge drinking–induced liver injury, oxidative stress, inflammatory response

## Abstract

*Panax ginseng* C.A. Meyer (ginseng) is an edible and traditional medicinal herb, which is reported to have a wide range of biological activity and pharmaceutical properties. There were more studies on ginsenoside and polysaccharides, but fewer on ginseng oligopeptides (GOPs), which are small molecule oligopeptides extracted from ginseng. The present study was designed to investigate the effects and underlying mechanism of ginseng oligopeptide (GOPs) on binge drinking-induced alcohol damage in rats. Sprague Dawley rats were randomly assigned to six groups (*n* = 10), rats in normal control group and alcohol model group was administered distilled water; rats in four GOPs intervention groups (at a dose of 0.0625, 0.125, 0.25, 0.5 g/kg of body weight, respectively) were administered GOPs once a day for 30 days. Experiment rats were intragastrically administered ethanol at a one-time dose of 7 g/kg of body weight after 30 days. The liver injury was measured through traditional liver enzymes, inflammatory cytokines, expression of oxidative stress markers, and histopathological examination. We found that the GOPs treatment could significantly improve serum alanine aminotransferase and aspartate aminotransferase, plasma lipopolysaccharide, and inflammatory cytokine levels, as well as the oxidative stress markers that were altered by alcohol. Moreover, GOPs treatment inhibited the protein expression of toll-like receptor 4, and repressed the inhibitor kappa Bα and nuclear factor-κB p65 in the liver. These findings suggested that GOPs have a significant protective effect on binge drinking-induced liver injury, and the mechanism possibly mediated by the partial inhibition of lipopolysaccharide—toll-like receptor 4-nuclear factor-κB p65 signaling in the liver.

## 1. Introduction

Binge drinking is defined by the National Institute on Alcohol Abuse and Alcoholism (NIAAA) as episodic excessive drinking of alcohol in a short period of time and elevating blood alcohol concentration (BAC) to 0.08% or higher, commonly means consuming more than four (women) or five (men) number of alcoholic beverages on one occasion [1,2]. The drinking pattern is now very popular among young populations, leading to increasing the risk for many social negative events and becoming a serious public health issue, including injuries, violence, suicide, and can induce systemic damage to multiple organs causing fatty liver, hypertension, gastrointestinal diseases, neuronal damage, and so on [3,4]. Globally, alcohol consumption accounts for 6.8% and 2.2% of age-standardized deaths among men and women, with a disproportionate effect on young people [5]. Most ingested ethanol is metabolized in the liver; excess alcohol consumption is a major cause of preventable liver disease worldwide. Many binge drinkers are susceptible to suffer from advanced alcoholic liver diseases (ALD), including a wide spectrum of hepatic lesions, such as fatty liver, hepatic fibrosis, and alcoholic hepatitis [5,6]. Unlike the health issues that are caused by chronic alcoholic consumption have been in-depth researched, less attention has been paid to health effects associated with acute alcoholism; especially liver consequences were often ignored [1,2]. Therefore, exploring underlying mechanisms, effective therapy, and agents for protecting alcoholic liver injury is of great importance.

The mechanism underlying the development of alcohol-induced liver damage remains unclarified, increasing evidence indicated that oxidative stress and inflammatory play a critical etiologic role in the development of ethanol-mediated liver injury, including hepatocyte dysfunction, apoptosis, and fibrosis, etc. [6,7,8,9]. Excessive ethanol consumption induce the accumulation of a variety of free radicals in the liver, reactive oxygen species (ROS), and reactive nitrogen species (RNS), including superoxide, hydrogen peroxide, lipid peroxides, nitric oxide, and peroxynitrite [2,7,10]. In turn, the overproduction of free radicals exceeded the ability of oxidative metabolism and destroyed the redox balance of the liver, which increased the consumption of antioxidant substances, inhibited the activity of superoxide dismutase (SOD), and decreased the level of antioxidant enzymes, especially glutathione (GSH). Thus, it leads to a series of pathological damage in the liver, induces lipid peroxidation of cellular membranes and oxidation of protein and DNA, which result in hepatocyte injury, inflammation, ischemia, fibrosis, necrosis, and apoptosis [2,11,12]. In previous studies, diets that were enriched with saturated fatty acids or medium chain triglycerides effectively against lipid peroxidation and pathological changes of ethanol-induced liver damage [13]. Moreover, dietary supplementation of antioxidants like vitamin E, superoxide dismutase, GSH precursors can effectively prevent early alcohol-induced liver injury [10]. Alcohol consumption also increases gut-derived bacterial endotoxin (lipopolysaccharide, LPS) and disrupts intestinal barrier functions that inducing endotoxin to into the systemic circulation and entering the liver tissue through the portal vein [6]. Previous studies demonstrated that LPS could be responsible for the development of ethanol-induced hepatic lesions. Kupffer cell activation by endotoxin via Toll-like receptor 4 (TLR4) and its coreceptors, CD14 and MD2, causing the rapid activation of the transcription factor nuclear factor κB (NF-κB) and then leading to enhance expression of several proinflammatory cytokines, such as tumor necrosis factor α (TNF-α), interleukin (IL)-6 and interleukin (IL)-1 [14,15,16,17]. Indeed, these results suggest that inhibition of these mediators like LPS and NF-κB reduced production of proinflammatory molecules and delayed the pathogenesis of alcoholic liver injury [14]. Therefore, inhibiting oxidative stress and inflammation would be effective in preventing the hepatotoxicity, and many studies have revealed that increasing dietary supplement with antioxidant and anti-inflammatory substances is likely a potential therapy for ALD [6,7].

*Panax ginseng* C.A. Meyer (ginseng) is a traditional medicinal herb that has been widely used as a restorative medicine for thousands of years, which helps to strength body resistance to stress, trauma, anxiety, and fatigue [18,19,20]. In 2012, ginseng and its products have been approved as new resources food by Ministry of Public Health of China [21]. Ginseng contains numerous active constituents, such as ginsenoside, polysaccharides, amino acids, and peptides. Owing to these numerous bioactive components, ginseng exhibit extensive pharmacological functions, including anti-oxidative activities, anti-hypoxia effect, anti-fatigue activity, immunoregulatory activity, hypolipidemic capacity, normalizing the human metabolic system, etc. [19,22,23]. With the advances of biochemistry and molecular biological techniques, various bioactive substances were extracted and discussed. Ginseng oligopeptides (GOPs), which are general name for small molecule oligopeptides isolated from ginseng with high bioavailability and absorption features. Previous studied showed that GOPs have numerous potential physiological functions, such as improving hyperglycemia [21], regulate innate and adaptive immune responses [22], enhancing stamina and relieve physical stress and fatigue [19], and attenuating irradiation-induced hematopoietic, gastrointestinal, and oxidative injury [24] Based on these reports, we hypothesized that the GOPs could have potential to against acute ethanol-induced liver injury mediated by redox imbalance and inflammation induced by endotoxin-mediated NF-κB activation. Therefore, this study aimed to investigate the possible protective effects of GOPs ameliorate binge drinking-induced liver injury and its underlying mechanism in a rat model of binge drinking.

## 2. Materials and Methods

### 2.1. Preparation and Identification of GOPs

The GOPs sample was provided by Jilin Taigu Biological Engineering Co., Ltd. (Jilin, China). It was extracted from the roots of *Panax ginseng* C.A. Meyer by enzymatic hydrolysis, which was planted in Jilin province, China. In brief, ginseng roots were cleansed, minced, homogenized in distilled water, and treated by complex protease (3000 U/g protein) at 40 °C for 3 h after adjusting the pH to 8.0 by sodium hydroxide. Next, nanofiltration, cryoconcentration, decolorization, purification, and spray drying were performed to obtain GOPs powders [19,21,22]. 

After purification by high-performance liquid chromatography (HPLC, Waters Corporation, Milford, MA, USA) using a Phenomenex C18 column (10 mm × 250 mm), the sample was measured by LDI-1700 matrixassisted laser desorption ionization time-of-flight mass spectrometer (MALDI-TOF-MS, Liner Scientific Inc., Reno, NV, USA). The identification results showed that 95.42% of GOPs had a molecular weight between 180 and 1000 Dalton. Amino acid accounted for 3.94%, which was further analyzed by an automatic amino acid analyzer (H835-50, Hitachi, Tokyo, Japan). The amino acid composition is shown in Table 1 [19,24].

### 2.2. Chemicals and Reagents

Ethanol was of analytical grade (Beijing Chemical Company, Beijing, China). Assay kit used for the determination of alanine aminotransferase (ALT), aspartate aminotransferase (AST), total cholesterol (TC), triglyceride (TG), high-density lipoprotein cholesterol (HDL-C), low-density lipoprotein cholesterol (LDL-C), total proteins (TP), globulin (GLB), albumin (ALB), globulin (GLB), creatinine (CR), and blood urea nitrogen (BUN) in the serum of rats were purchased form Yingkexinchuang Science and Technology Ltd. (Macau, China). The detection kits of superoxide dismutase (SOD), glutathione peroxidase (GSH-Px), malondialdehyde (MDA), glutathione (GSH) levels, very low density lipoprotein (VLDL), tumor necrosis factor α (TNF-α), interleukin (IL)-6, interleukin (IL)-1β and lipopolysaccharide (LPS), and bicinchoninic acid (BCA) protein assay kit were purchased from Beyotime Institute of Biotechnology (Beijing, China). The primary antibodies against rabbit toll-like receptor 4 (TLR4), CD14, nuclear factor-κB p65 (NF-κB p65), and inhibitor kappa Bα (IκBα) were purchased from Abcam (Cambridge, UK). All of the reagents used in this study were of analytical grade. 

### 2.3. Animals and Experimental Design

Sixty male Sprague–Dawley (SD) rats (weighing 180–220 g) were obtained from the Animal Service of Health Science Center, Peking University (Laboratory animal license No.: syxk (Jing) 2011-0039, laboratory animal production license No.: scxk (Beijing) 2011-0012). Rats were housed three per cage in a filter-protected, air-conditioned room with constant temperature (21–25 °C), relative air humidity of 50–60%, and a 12 h:12 h dark cycle (lights on at 07.30–19.30 h). All of the rats had free access to standard food (American Institute of Nutrition Rodent Diets-93G (AIN-93G diet) and water. The experiment was reviewed and approved by the Institutional Animal Care and Use Committee of Peking University, and animal treatments and experimental procedures were carried out strictly in accordance with the Principle of Laboratory Animal Care (National Institutes of Health publication NO.85-23, revised 1985) and the guidelines of the Peking University Animal Research Committee (www.lab.pku.edu.cn).

After acclimatization for one week, SD rats were randomly divided into six experimental groups (*n* = 10): normal control group, ethanol group, and four GOPs intervention groups at different doses (0.0625, 0.1250, 0.2500, 0.5000 g/kg body weight (BW), namely GOPs 1, GOPs 2, GOPs 3, GOPs 4, respectively). Rats in four GOPs groups were subjected to oral treatments once daily for 30 days by oral gavage, whereas the control group and ethanol group were intragastrically administered with distilled water. The doses setting were referred to the previous study in our lab [19,21,22]. During the experimental period, all groups were allowed free access to water and food, body weight and food consumption were measured every week. At day 30, rats were food restricted and were given only water to drink for 6 h after final administration. Then, animals were intragastrically administered a one-time dose of 50% ethanol (7 g/kg BW, namely 17.5 mL/kg), except for those in normal control group that received equivalent 0.9% saline. All that rats kept fasting for 16 h after alcoholic gavage, then subsequently anesthetized with diethyl ether. Blood sample was collected from femoral artery; serum was prepared by centrifugation at 3000 rpm at 4 °C for 15 min to detect enzymes and inflammatory cytokines. Livers were then obtained and preserved at −80 °C for further analysis.

### 2.4. Biochemical Assays and Enzyme-Linked Immunobsorbent Assay

The levels of ALT, AST, TC, TG, HDL-C, LDL-C, TP, GLB, ALB, CR, and BUN in the serum were measured by an automatic biochemical analyzer (Olympus Corporation, Tokyo, Japan). The activities of SOD, GSH-Px, GSH, MDA, VLDL in the liver and the levels of VLDL, TNF-α, IL-6, IL-1β, and LPS in serum were assayed by enzyme-linked immunosorbent assay (ELISA) kits according to the protocol provided by the manufacture.

### 2.5. Histopathological Exanination

For histopathological analysis, the liver tissues (*n* = 6 per group) were acquired from the same lobe were fixed in 10% (*v*/*v*) buffered formaldehyde for over 24 h, subsequently paraffin embedded liver sections (5 mm-thick), and then stained with hematoxylin-eosin (H&E). The slides were observed with an Olympus IX70 inverted microscope (Olympus, Tokyo, Japan), and representative images were presented.

The pathological change of the liver was recorded from one end of the slides and the whole tissue sections were continuously observed under light microscope. The hepatic fat accumulation in the liver was mainly observed and each liver section was assigned a score from Level I to IV. The scoring criteria is shown in Table 2.

### 2.6. Western Blot Analysis

Total proteins in liver cells were extracted using RIPA lysis buffer (1% Triton X-100, 1% deoxycholate, 0.1% in sodium dodecyl sulfate) with 1 mM phenylmethanesulfonyl fluoride (PMSF). Nuclear protein was extracted from the liver tissues and protein concentration was measured by the BCA procedure. Then, 20µg protein samples were separated by using sodium dodecyl sulfate-polyacrylamide gel electrophoresis and then transferred onto 0.45 µm (pore size) polyvinylidene fluoride membranes (EMD Millipore, Bedford, MA, USA). Membranes were blocked with 5% (*m*/*v*) skimmed milk at room temperature for 1 h. TLR4, CD14, and NF-κB antibodies diluted with 5% (*m*/*v*) bovine serum albumin at a ration of 1:200, IκBαa at 1:1000, β-Actin at 1:200 that was used to verify equal total protein, and then were placed at 4 °C for overnight. After washing with TBST three times, the membranes were incubated with secondary antibody goat anti-rabbit (at 1:5000) for 1 h at room temperature. The bands were detected using an enhanced chemiluminescence system (Applygen Technologies, Inc., Beijing, China) and were analyzed by Quantity One software. 

### 2.7. Statistical Analysiss

All data were analyzed by the SPSS software version 24 (SPSS Inc., Chicago, IL, USA). Values were presented as mean ± standard deviation (SD). Differences between groups were analyzed by the one-way analysis of variance (ANOVA) test with least significant difference (LSD) methods if the data were homogeneous. The Nonparametric Test (Kruskal-Wallis) was used for the histological examination comparison. Differences were considered to be statistically significant when *p* < 0.05.

## 3. Results

### 3.1. Effects of GOPs on Body Weight and Organ Index

As shown in Table 3, daily oral administrated of GOPs did not cause any death in the treated rats, and there was no significant changes in final body weight among the groups. The organ index was calculated as a ratio (%) of the organ weight (g) to body weight (g) [25]. The liver index was significantly higher in five alcohol-treated groups than that in the normal control group (*p* < 0.05), but no significant difference were observed in the kidney index among all groups.

### 3.2. Effects of GOPs on Serum Biochemical Parameters

Alanine aminotransferase (ALT) and aspartate aminotransferase (AST) levels are common pathological parameters used to evaluate hepatocyte damage [25]. As shown in Table 4, in comparison with the normal control group, serum levels of the ALT and AST were greatly increased in the alcohol group (*p* < 0.01), which suggested that alcohol has induced acute liver injury and the experimental model has been established successfully [5]. The activities of ALT and AST in four GOPs groups, which GOPs pretreatment for 30 days, were remarkably lower than alcohol group (*p* < 0.01), and there were no significant difference when compared with normal control group (*p* > 0.05). The results indicated that the pretreatment of GOPs could inhibit the impaired liver functions at some extent resulting from alcohol-induced toxicity.

The contents of total cholesterol (TC), high-density lipoprotein cholesterol (HDL-C), and Low-density lipoprotein cholesterol (LDL-C) were significantly decreased, triglyceride (TG) and very low density lipoprotein (VLDL) were significantly elevated in the ethanol group as compared with the normal control group (*p* < 0.05 or *p* < 0.01), indicated alcohol induced abnormal lipid metabolism in rats. However, the levels of TC, TG, HDL-C, and LDL-C in four GOPs intervention groups were no significant differences when compared with ethanol group (*p* > 0.05), whereas only the VLDL levels significantly decreased (*p* < 0.01). 

Serum total proteins (TP), globulin (GLB), albumin (ALB), globulin (GLB), and Creatinine (CR) levels were significantly decreased in alcohol and four GOPs treatment groups compared with the normal control group (*p* < 0.01), but no significant difference were found in all GOPs groups as compared with the ethanol group (*p* > 0.05). Albumin: globulin (A:G) ratio (except for GOPs 2, 0.1250 g/kg BW GOPs group) and alkaline phosphatase (ALP) contents in the alcohol group and four GOPs groups were higher than the normal control group (*p* < 0.01), and the activities of blood urea nitrogen (BUN); there were no significance differences in all seven groups (*p* > 0.05).

### 3.3. Histopathological Analysis

**Figure 1:**
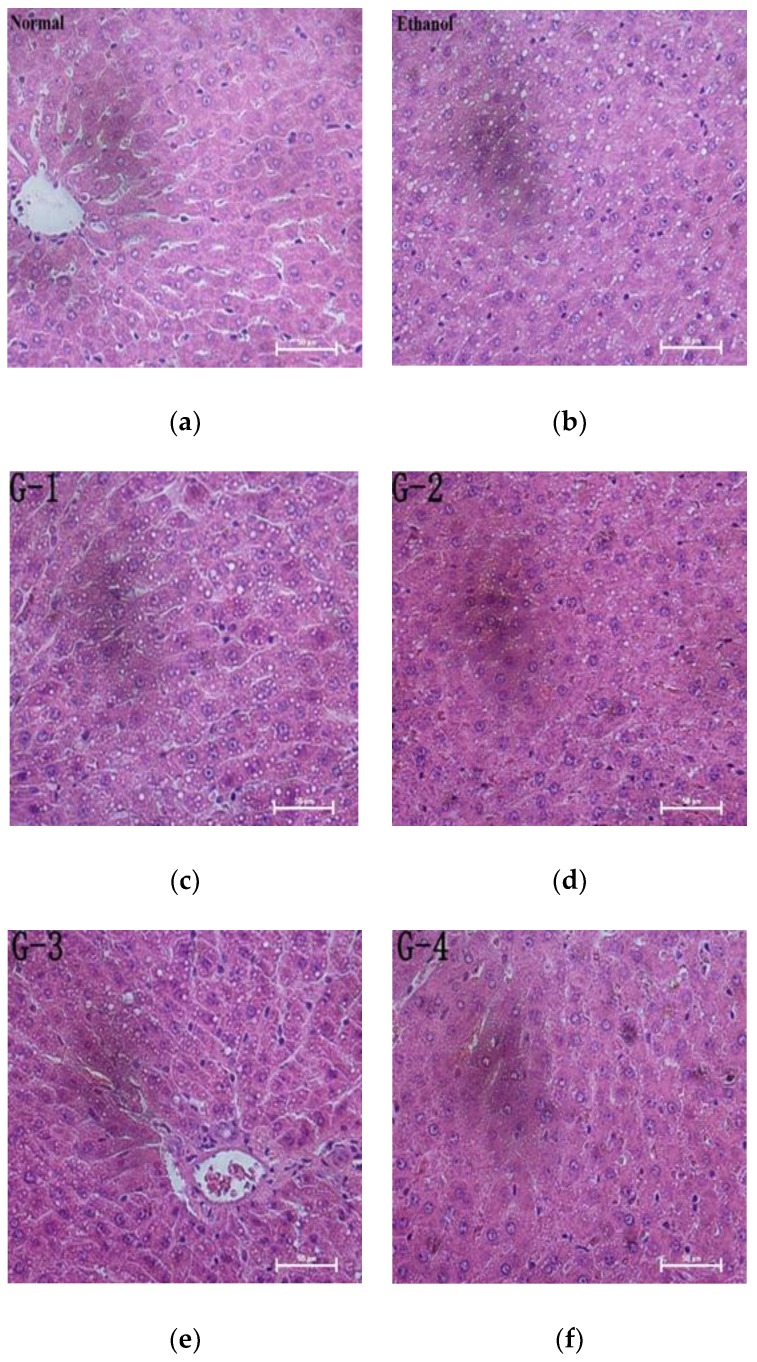
Effect of GOPs on liver histology in rats. Representative photomicrographs with H&E staining reveal histopathological changes of liver (400×). GOPs, small molecule oligopeptides isolated from ginseng; (**a** )normal group; (**b**) ethanol group; (**c**) G-1, 0.0625 g/kg BW GOPs group; (**d**) G-2, 0.1250 g/kg BW GOPs group; (**e**) G-3, 0.2500 g/kg BW GOPs group; (**f**) G-4, 0.5000 g/kg BW GOPs group.

Representative photomicrographs exhibiting liver pathology (H & E staining) from different groups are shown in Figure 1. The normal group had normal hepatic architecture, and there was no visible lesions (Figure 1a). However, in the ethanol group, the hepatocytes showed obvious steatosis and irregular arrangement, which confirmed the successful establishment of liver injury (Figure 1b). Pretreatment of GOPs groups exerted a protective effect against alcohol-induced damage, the hepatocyte steatosis of GOPs treatment groups were attenuated (Figure 1c–f), and more regular hepatic cords and hepatocytes with clear border were observed in the liver of rats in the GOPs 3 and 4 groups (Figure 1e,f, respectively). As shown in Table 5, hepatic steatosis were significantly aggravated in alcohol group compared with normal group by *Kr’uskal-Wallis* analyses (*p* < 0.05). Although there was no significant difference between four GOPs groups and alcohol group (*p* > 0.05), the pathological scores of level III in GOPs intervention groups were lower than the alcohol group and there was no level IV presented in GOPs groups. These results indicated that the distribution of hepatocytes with lipid droplets in GOPs groups might be reduced to a certain extent, and GOPs pretreatment might have potential activity to alleviate alcohol-induced hepatic steatosis. 

### 3.4. Effect of GOPs on Alcohol-Induced Oxidative Stress

**Figure 2:**
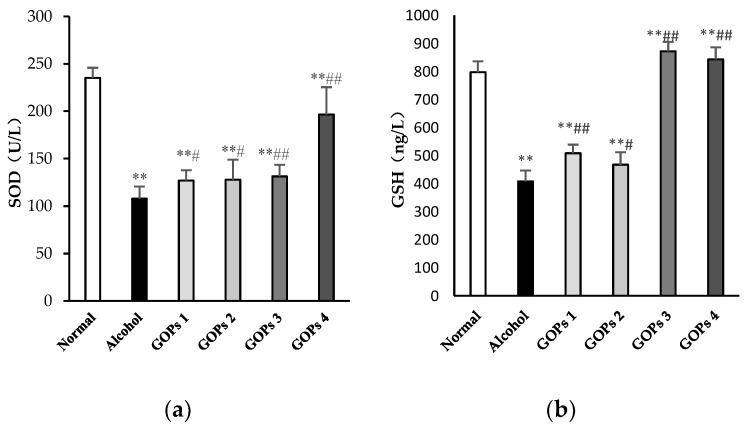

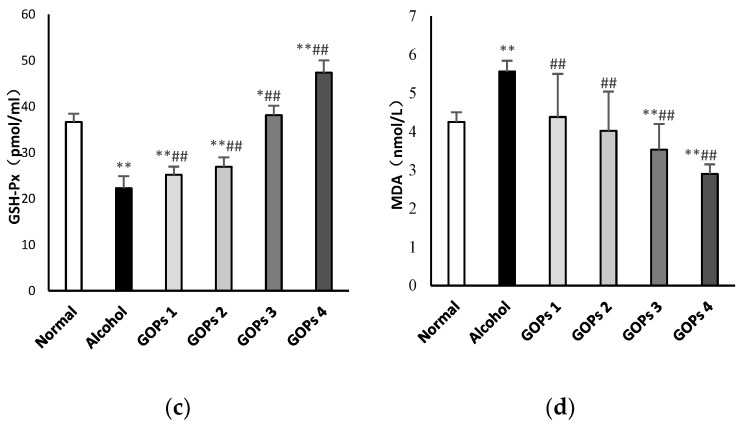
Effects of GOPs on hepatic SOD (**a**), GSH (**b**), GSH-PX (**c**), MDA (**d**) levels in alcohol-induced rats. Data represent the mean ± SD (*n* = 10). Significant differences were indicated by * *p* < 0.05 versus control group, ** *p* < 0.01 versus control group; ^#^ *p* < 0.05 versus alcohol group, ^##^ *p* < 0.01 versus alcohol group. SOD, superoxide dismutase; GSH, reduced glutathione; GSH-Px, glutathione peroxidase; MDA, malondialdehyde. GOPs, small molecule oligopeptides isolated from ginseng; GOPs 1 to 4 refer to that mice were treated with GOPs at 0.0625, 0.1250, 0.2500, 0.5000 g/kg for 30 days, respectively.

Numerous studies have been demonstrated that oxidative stress has been strongly related to ethanol-induced liver injury and ALD pathogenesis [10,25]. In order to investigate the effect of GOPs on alcohol-induced oxidative stress, the activity of superoxide dismutase (SOD), reduced glutathione (GSH), glutathione peroxidase (GSH-Px), and malondialdehyde (MDA) in the liver tissues of rats were examined. As shown in Figure 2, when compared with those of normal control rats, the activities of SOD, GSH and GSH-Px in the alcohol group were significantly decreased, while the MDA contents were remarkably increased (*p* < 0.01), indicated that the hepatocyte was significantly damaged by oxidative stress after alcohol exposure. However, when compared with the alcohol control group, the intervention of GOPs exhibited protection against alcohol-induced SOD, GSH, and GSH-Px depletion and MDA accumulation in the liver tissues of rats (all GOPs groups: *p* < 0.01). 

### 3.5. Effects of GOPs on Serum Lipopolysaccharide and Inflammatory Cytokine Levels

**Figure 3:**
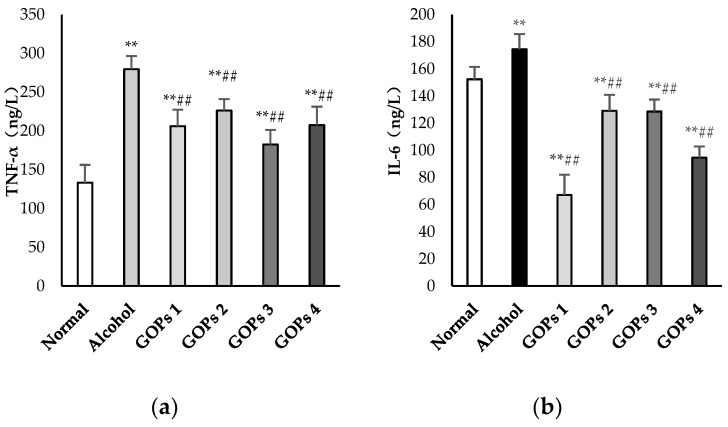

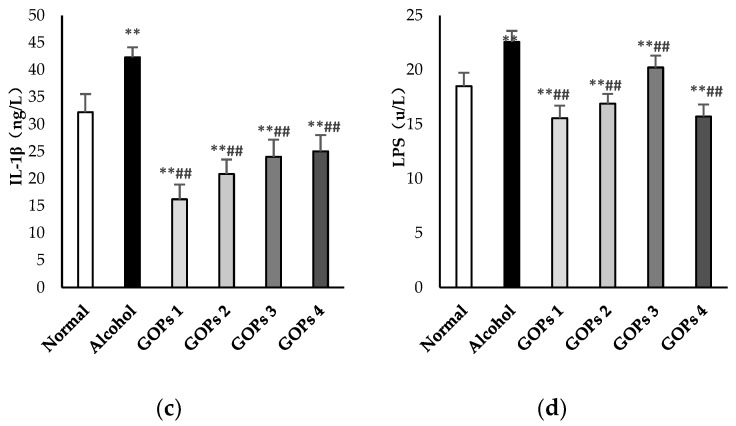
Effects of GOPs on inflammatory cytokine and lipopolysaccharide levels in rats. Values are presented as the mean ± standard deviation of 10 rats per group. (**a**), tumor necrosis factor-α(TNF-α); (**b**), interleukin (IL)-6(IL-6); (**c**) interleukin (IL)-1β(IL-1β); and, (**d**), lipopolysaccharide (LPS). * *p* < 0.05 versus control group, ** *p* < 0.01 versus control group; ^#^ *p* < 0.05 versus alcohol group, ^##^ *p* < 0.01 versus alcohol group. GOPs, small molecule oligopeptides isolated from ginseng; GOPs 1 to 4 refer to administration of GOPs at a dose of 0.0625, 0.1250, 0.2500, 0.5000 g/kg, respectively.

As shown in Figure 3, serum tumor necrosis factor α (TNF-α), interleukin (IL)-6, interleukin (IL)-1β, and lipopolysaccharide (LPS) levels after ethanol exposure were significantly higher than those in the normal control group (*p* < 0.01). The results suggested that a one-time dose of 50% ethanol (17.5 mL/kg) oral gavage induced the excessive reaction of inflammatory cells and intestinal endotoxemia (IETM) in rats. However, in comparison with the alcohol group, the contents of serum TNF-α, IL-6, IL-1β, and LPS in the four GOPs groups were greatly reduced, and the difference was statistically significant (*p* < 0.01).

### 3.6. Effect of GOPs on the TLR4, CD14, NF-κB p65 and Iκbα Expression in Liver Tissues 

**Figure 4:**
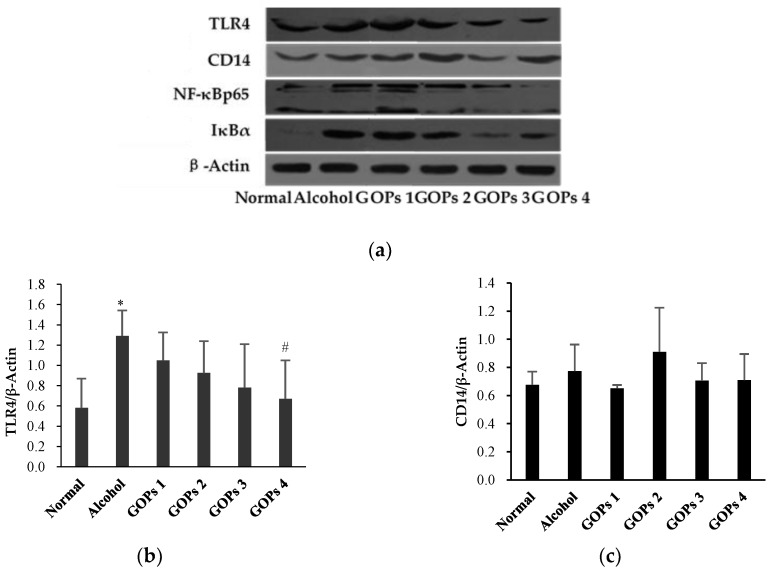

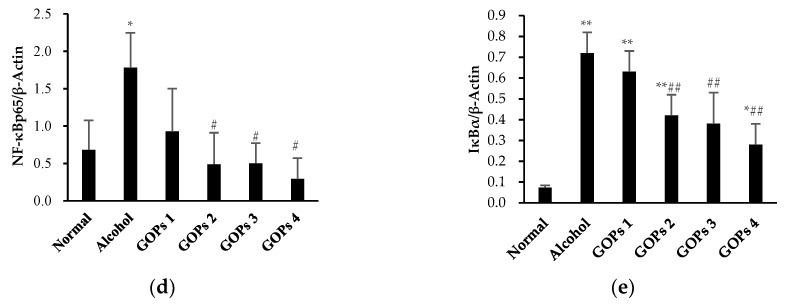
Effect of GOPs on the protein expression of TLR4, CD14, NF-κB p65, and IκBα in the liver of rats from each group. Values of all proteins were normalized to those of β-actin as the control. (**a**), β-actin; (**b**), toll-like receptor 4 (TLR4); (**c**), CD14; (**d**), nuclear factor-κB p65 (NF-κB p65); and, (**e**), inhibitor kappa Bα (IκBα). Values are expressed as mean ± standard deviation of at least three independent experiments. * *p* < 0.05 versus control group, ** *p* < 0.01 versus control group; ^#^ *p* < 0.05 versus alcohol group, ^##^ *p* < 0.01 versus alcohol group. GOPs, small molecule oligopeptides isolated from ginseng; GOPs 1 to 4 refer to administration of GOPs at a dose of 0.0625, 0.1250, 0.2500, 0.5000 g/kg, respectively.

The protein expression of toll-like receptor 4 (TLR4), CD14, nuclear factor-κB p65 (NF-κB p65), and inhibitor kappa Bα (IκBα) in the liver of each group were detected by western blot analysis. As shown in Figure 4, the expression of TLR4, NF-κB p65, and IκBα in the alcohol group were increased significantly when compared with the normal control group (*p* < 0.05 for TLR4 and NF-κB p65, *p* < 0.01 for IκBα), but CD14 levels were no significant differences among all groups (*p* > 0.05). However, these protein expression levels were markedly reversed at some extent by GOPs. TLR4 in the GOPs 4 group, NF-κB p65 and IκBα in the GOPs2 to GOPs4 groups were reduced significantly as compared with those of the alcohol group (*p* < 0.05 for TLR4 and NF-κB p65, *p* < 0.01 for IκBα).

## 4. Discussion

Excessive alcohol intake facilitates liver damage, which is a major risk factor for both death and the burden of disease and injury worldwide [5,6]. Most studies of alcoholic liver damage have focused on the effects of chronic alcohol exposure. However, binge drinking is more common than chronic alcoholism. Thus, the exploration of the acute alcohol consumption induced liver injury is warranted. It has been well documented that fatty accumulation, oxidative stress and inflammation are critical in the development and progression of alcoholic liver injury [6,26]. *Panax ginseng* C.A. Meyer (ginseng) has been proved to be an effective natural substance for accelerating alcohol metabolism and enhancing liver function, which has strong effects of antioxidant, anti-inflammatory, and improvement of immunity [27,28,29,30]. Much attention has been focused the ginsenoside, and polysaccharides presented in ginseng. However, there has been rarely studies in investigating the effects of GOPs, which include small molecule oligopeptides isolated from *Panax ginseng* C.A. Meyer. In the present study, the results showed that GOPs supplementation enhanced antioxidant capacity, suppressed inflammatory response, and improved the dysfunction of liver in binge drink rats. Additionally, it was found that the effect of GOPs might be mediated by the attenuation of the LPS-TLR4-NF-κB signaling pathway.

Most studies of acute alcoholic liver damage have been performed in rodent models, which often divided into three types: single binge, intermittent heavy drinking and chronic alcohol exposure followed by episodes of binge, but there is no generally accepted method to date [26,31,32]. It was found that ethanol was administered at doses of 6–7 g/kg in rodents that produced peak blood ethanol concentrations that are similar to those that might be present in humans during acute alcoholism, which took into account differences in alcohol metabolism between rodents and humans [26,33,34]. Various factors cause abnormal lipid accumulation in lipid droplets in the liver, known as hepatic steatosis, which is the first and most common hepatic change induced by alcohol consumption [26,35,36]. Elevated levels of serum ALT and AST have been considered as sensitive indicators of alcoholic liver injury [8,37,38]. In the present study, we developed the binge drink-induced liver injury model in SD rats by gavage 7 g per kg body weight after supplementation with GOPs for 30 days. The SD rats showed hepatocyte steatosis, higher serum ALT, AST levels, and liver index after alcohol treatment, which were similar to those of Xia T, Bukong T N, Chang B, and Chen P et al. [6,39,40,41]. These changes suggested that our protocol resulted in binge drink-induced liver injury, although no inflammatory or apoptotic changes were observed in liver histopathology. The results indicated that GOPs pretreatment alleviated the accumulation of lipid droplets to some extent and remarkably reversed the elevation of serum ALT and AST levels in rats, which suggested that GOPs exerted a protective effect against alcohol-induced liver dysfunction in the present study.

The pathogenesis of binge drink-induced liver injury involves oxidative stress and damages, that is, excessive generation of ROS and an oxidant and antioxidant imbalance under the stimulation of alcohol [6,9]. Importantly, oxidative stress and the antioxidant system appear to be crucial modulators of various processes involved, such as cellular degradation of lipids, proteins and DNA, modulation of functional signaling pathways, including the mitogenactivated protein kinase (MAP kinase), and nuclear factor κ-B (NFκB) pathways, all of which play important roles in the development and progression of liver injury [9]. Actually, alcohol induced oxidative stress is achieved primarily by increasing cellular oxidants (e.g., O_2_, H_2_O_2_) and lipid peroxidation products (e.g., MDA) and reducing antioxidants (e.g., SOD, GSH) in the liver [9,25,42]. For instance, SOD is a principal endogenous antioxidant enzyme to attenuate the damage induced by excessive oxidative stress, which by scavenging free radicals and their metabolites and even accelerating its clearance [25]. GSH is a low molecular weight scavenger that clears O_2_, H_2_O_2_, and protects DNA, proteins and other cofactors from oxidative damage. While GSH-Px can specifically catalyze the reaction of GSH to hydrogen peroxide, to protect the integrity of the structure and function of cell membrane [7]. Meanwhile, MDA is the main degradation products of lipid peroxidation; in addition, as a crucial indicator to reflect the severity of free radical attacks on the body cells indirectly [25]. Consequently, maintaining suitable levels of SOD, GSH, GSH-Px, and MDA are regarded as essential for preventing and reducing oxidative damage induced by alcohol-mediated ROS and radicals [7,25]. In this study, the results showed that alcohol exposure significantly decreased the levels of SOD, GSH and GSH-Px, while increased the MDA contents in the hepatocyte of rats. However, pretreatment of GOPs before ethanol exposure reversed the depletion of these antioxidant enzymes and the elevation of MDA contents. Based on the results above, it can be concluded that GOPs have the capacity to restore binge drinking-induced liver injury, partly due to the inhibition of oxidative stress by suppressing lipid peroxidation and enhancing antioxidant defense.

Binge alcohol drinking in humans and experiment models is characterized by increased serum endotoxins (i.e., LPS) and over production of various proinflammatory cytokines [40,43]. Considerable evidence has accumulated in support of that the increased circulating bacterial endotoxin plays an important role in early alcohol-induced liver injury in both healthy volunteers and alcoholic with ALD, as well as in experimental models of acute alcohol exposure [14,26,44,45]. Acute excessive alcohol exposure increases gut derived LPS in the portal circulation, a component of the gram-negative bacterial wall, which binds to the LPS-binding protein, and then transfers initially to the CD14 receptor and ultimately interacts with TLR4 on Kupffer cells [15,46,47]. The activation of TLR-4, which is a transmembrane protein that mediates LPS-induced signal transduction, activates two signaling pathway the myeloid differentiation factor 88 (MYD88)-dependent pathway and the TIR-domain-containing adapter inducing interferon-β (TRIF)/IRF-3 signaling pathway via the recruitment of adaptor molecules [15,44,48]. NF-κB could be activated by both two pathways, although the significance of these two pathways in ALD are yet to be further evaluated. Generally, NF-κB interacts with IκB to keep it inactive in the cytoplasm, unless various stimuli lead it return to phosphor-IκB and then IκB degrades, which allows NF-κB to translocate to the nucleus and induced the expression of its target genes [25,49]. The activation of NF-κB leads to the release of various proinflammatory cytokines, such as TNF-a, IL-6, and IL-1β, thereby contributing to hepatocyte dysfunction [25,43,48]. TNF-α is an important pro-inflammatory cytokine in the development of ALD, which is closely related to inflammatory response, lipid metabolism, and cell death [49]. IL-6 is considered as another important pro-inflammatory factor in ALD. IL-1 produces various metabolic events of acute liver injury symptom and involves the development of hepatic steatosis [43]. In the present study, the results showed that the LPS, TNF-a, IL-6, and IL-1β levels were significantly increased in alcohol-administrated rats, which was consistent with the results that were found in other related studies [40,45]. These results indicated the involvement of the inflammatory response in acute-induced liver injury. However, the levels of LPS and proinflammatory cytokines were markedly restored by GOPs pretreatment, which indicated the protective capacity of GOPs against binge drink-induced inflammation. Additionally, our results confirmed that alcohol exposure significantly up-regulated the protein expression of TLR4 and its downstream NF-κB p65 and IκBα. Notably, the change of these proteins was improved by GOPs administration, which provides new insight into the action of GOPs on alcoholic liver injury. Taken together, these results indicate that GOPs can inhibit alcohol-induced liver inflammation via inhibiting the LPS-TLR4-NF-κB signal pathway in rats. 

## 5. Conclusions

The current study demonstrated that GOPs administration could partially, but not completely, alleviate lipid peroxidation and improve serum alanine aminotransferase and aspartate aminotransferase, plasma lipopolysaccharide, and inflammatory cytokine levels, as well as the oxidative stress markers altered by alcohol, thereby ameliorating binge drink-induced liver injury of rats. Moreover, GOPs treatment inhibited the protein expression of TLR 4, IκBα, and NF-κB p65 in the liver, which indicated that the attenuation of proteins that are involved in LPS-TLR4-NF-κB pathway might be one of the possible mechanisms involved in the liver protective effects of GOPs. These data provide important prospects for the application of GOPs as a natural functional food against alcoholic liver injury. Further research is necessary to elucidate which one or both of the two pathways, the MyD88-dependent pathway and TRIF-dependent pathway, is possibly involved in the protective mechanisms, and determine the optimal dose of GOPs supplementation for further evaluation in humans. 

## Figures and Tables

**Table 1:** Amino acid composition of ginseng oligopeptides (GOPs).

Amino Acid	Amino Acid Composition of GOPs (g/100 g)
Asp	0.19
Glu	0.12
Ser	0.02
His	0.06
Gly	0.02
Thr	0.05
Arg	2.26
Ala	0.13
Tyr	0.09
Cys	0.01
Val	0.06
Met	0.02
Phe	0.09
Ile	0.04
Leu	0.08
Lys	0.06
Pro	0.65

GOPs, small molecule oligopeptides isolated from ginseng.

**Table 2:** The scoring criteria of hepatic steatosis.

The Extent of Hepatic Fat Accumulation	Level
Lipid droplets in liver cells scattered, scarce and normal	0
Hepatocytes with lipid droplets do not exceed 1/4 of the entire picture	I
Hepatocytes with lipid droplets do not exceed 1/2 of the entire picture	II
Hepatocytes with lipid droplets do not exceed 3/4 of the entire picture	III
Lipid droplets affected almost the entire liver tissue	IV

**Table 3:** Effects of GOPs on body weight and organ index in rats.

Groups	Dosage (g/kg BW)	Body Weight (g)		Liver Index (%)	Kidney Index (%)
		Initial	Final		
Normal		175.47 ± 9.97	397.47 ± 13.57	2.63 ± 0.22	0.61 ± 0.03
Alcohol		172.25 ± 7.03	391.30 ± 16.97	2.93 ± 0.24 *	0.61 ± 0.03
GOPs 1	0.0625	172.60 ± 7.54	392.93 ± 17.24	3.03 ± 0.31 *	0.63 ± 0.06
GOPs 2	0.1250	174.13 ± 9.72	387.65 ± 15.60	3.00 ± 0.25 *	0.65 ± 0.06
GOPs 3	0.2500	173.33 ± 7.83	392.20 ± 13.39	2.96 ± 0.30 *	0.63 ± 0.06
GOPs 4	0.5000	170.93 ± 7.15	398.07 ± 17.30	2.99 ± 0.27 *	0.63 ± 0.04

The data were analyzed for significance of difference by one-way analysis of variance (ANOVA) test, expressed as means ± SD; *n* = 10 for each group. Mean values were significantly different from those of the normal control group, * *p* < 0.05. GOPs, small molecule oligopeptides isolated from ginseng. BW, body weight.

**Table 4:** Effects of GOPs on aminotransferase, lipid, protein, urea nitrogen, and creatinine in the serum of rats.

Parameters	Normal	Alcohol	GOPs 1	GOPs 2	GOPs 3	GOPs 4
ALT (U/L)	52.50 ± 9.76	74.40 ± 15.24 **	58.00 ± 12.36 ^##^	54.70 ± 7.85 ^##^	60.00 ± 9.17 ^##^	57.80 ± 9.04 ^##^
AST (U/L)	211.50 ± 37.21	285.50 ± 47.17 **	230.60 ± 24.64 ^##^	213.50 ± 27.46 ^##^	238.00 ± 41.19 ^##^	232.40 ± 32.10 ^##^
TC (mmol/L)	1.65 ± 0.28	1.29 ± 0.12 *	1.54 ± 0.49	1.44 ± 0.18	1.43 ± 0.26	1.45 ± 0.30
TG (mmol/L)	0.56 ± 0.14	1.24 ± 0.20 **	1.02 ± 0.4	1.04 ± 0.58	0.64 ± 0.19 ^##^	0.73 ± 0.16 ^##^
HDL-C (mmol/L)	1.58 ± 0.17	1.12 ± 0.11 **	1.13 ± 0.40	1.25 ± 0.17 *	1.18 ± 0.31 *	1.23 ± 0.19 *
LDL-C (mmol/L)	0.40 ± 0.07	0.23 ± 0.04 **	0.22 ± 0.08 **	0.20 ± 0.05 **	0.21 ± 0.05 **	0.19 ± 0.03 **
VLDL (μg/mL)	130.10 ± 13.02	203.38 ± 15.66 **	153.06 ± 13.29 **^##^	89.00 ± 13.63 **^##^	107.53 ± 16.57 **^##^	124.35 ± 19.88 ^##^
TP (g/L)	67.64 ± 3.58	57.34 ± 4.93 **	54.99 ± 6.44 **	59.35 ± 7.65 **	54.22 ± 7.63 **	56.92 ± 5.49 **
ALB (g/L)	38.21 ± 1.99	34.02 ± 2.29 **	33.33 ± 3.01 **	34.28 ± 3.59 **	32.05 ± 3.27 **	33.68 ± 2.45 **
GLB (g/L)	29.43 ± 1.99	23.31 ± 2.85 **	21.66 ± 3.51 **	25.07 ± 4.17 **	22.17 ± 4.48 **	23.25 ± 3.13 **
A:G ratio	1.30 ± 0.07	1.47 ± 0.12 **	1.56 ± 0.13 **	1.38 ± 0.11 #	1.47 ± 0.15 **	1.46 ± 0.10 **
ALP (U/L)	114.66 ± 22.14	176.07 ± 37.57 **	213.01 ± 92.34 **	203.89 ± 72.39 **	237.45 ± 63.19 **^##^	185.08 ± 38.53 **
CR (umol/L)	35.00 ± 2.51	31.66 ± 3.05 *	29.70 ± 3.82 **	29.36 ± 3.75 **	29.40 ± 2.60 **	30.85 ± 4.83 **
BUN (mmol/L)	4.70 ± 0.68	4.69 ± 0.97	5.33 ± 0.61	4.74 ± 0.82	4.88 ± 1.07	4.88 ± 0.54

Values represent as the mean ± SD (*n* = 10). * *p* < 0.05 versus normal control group, ** *p* < 0.01 versus normal control group; ^#^ *p* < 0.05 versus alcohol group, ^##^ *p* < 0.01 versus alcohol group. ALT, alanine aminotransferase; AST, aspartate aminotransferase; TC, total cholesterol; TG, triglyceride; HDL-C, high-density lipoprotein cholesterol; LDL-C, low-density lipoprotein cholesterol; VLDL, very low density lipoprotein; TP, total protein; ALB, albumin; GLB, globulin; A:G ratio, albumin: globulin; ALP, Alkaline phosphatase; CR, Creatinine; BUN, blood urea nitrogen. GOPs, small molecule oligopeptides isolated from ginseng; GOPs 1–4 refer to 0.0625, 0.1250, 0.2500, 0.5000 g/kg BW GOPs intervention group, respectively.

**Table 5:** Score result of the pathological changes in liver of rats.

Groups	Dosage (g/kg BW)		Steatosis Grade				Average Rank
		0	I	II	III	IV	
Normal		5	1	0	0	0	6.42
Alcohol		0	0	1	3	2	30.42 *
GOPs 1	0.0625	0	1	3	2	0	23.00
GOPs 2	0.1250	1	2	2	1	0	17.50
GOPs 3	0.2500	1	2	1	2	0	18.92
GOPs 4	0.5000	2	2	1	1	0	14.75

Code of point (*n* = 6). The *Kruskal-Wallis* test was used for the histological examination comparison. * *p* < 0.05 versus control group. Level 0 calculated 0 mark; level I calculated 1 mark; level II calculated 2 mark; level III calculated 3 mark; level IV calculated 4 mark. GOPs, small molecule oligopeptides isolated from ginseng.
